# Stabilization by Nano Spray Dryer of Pioglitazone Polymeric Nanosystems: Development, In Vivo, Ex Vivo and Synchrotron Analysis

**DOI:** 10.3390/pharmaceutics13111751

**Published:** 2021-10-20

**Authors:** Marcelle Silva-Abreu, Esther Miralles, Christina S. Kamma-Lorger, Marta Espina, María Luisa García, Ana Cristina Calpena

**Affiliations:** 1 Department of Pharmacy, Pharmaceutical Technology and Physical Chemistry, Faculty of Pharmacy and Food Sciences, University of Barcelona, 08028 Barcelona, Spain; m.espina@ub.edu (M.E.); marisagarcia@ub.edu (M.L.G.); anacalpena@ub.edu (A.C.C.); 2 Institute of Nanoscience and Nanotechnology (IN2UB), University of Barcelona, 08028 Barcelona, Spain; 3 CCiTUB (Scientific and Technological Centers), University of Barcelona, 08028 Barcelona, Spain; emiralles@ub.edu; 4 BioSAXS ANSTO Australian Synchrotron, Clayton, VIC 3168, Australia; ckamma@ansto.gov.au

**Keywords:** PLGA-PEG, pioglitazone, nanoparticles, spray drying, cornea, synchrotron, SAXS

## Abstract

Pioglitazone-loaded PLGA-PEG nanoparticles (NPs) were stabilized by the spray drying technique as an alternative to the treatment of ocular inflammatory disorders. Pioglitazone-NPs were developed and characterized physiochemically. Interaction studies, biopharmaceutical behavior, ex vivo corneal and scleral permeation, and in vivo bioavailability evaluations were conducted. Fibrillar diameter and interfibrillar corneal spacing of collagen was analyzed by synchrotron X-ray scattering techniques and stability studies at 4 °C and was carried out before and after the spray drying process. NPs showed physicochemical characteristics suitable for ocular administration. The release was sustained up to 46 h after drying; ex vivo corneal and scleral permeation profiles of pioglitazone-NPs before and after drying demonstrated higher retention and permeation through cornea than sclera. These results were correlated with an in vivo bioavailability study. Small-angle X-ray scattering (SAXS) analysis did not show a significant difference in the organization of the corneal collagen after the treatment with pioglitazone-NPs before and after the drying process, regarding the negative control. The stabilization process by Nano Spray Dryer B-90 was shown to be useful in preserving the activity of pioglitazone inside the NPs, maintaining their physicochemical characteristics, in vivo bioavailability, and non-damage to corneal collagen function after SAXS analysis was observed.

## 1. Introduction

During the last decades many synthetic and natural polymers have been studied in nanomedicine as drug delivery systems [1,2,3], and today their use is well established. Polymeric nanoparticles (NPs) are systems that improve drug delivery in tissues and organs by modifying drug release behavior and sustainability, thus increasing the drug bioavailability across biological membranes [4].

Polylactic-co-glycolic acid (PLGA) is a synthetical polymer with biocompatibility and resorbability properties, and it is approved by the US Food and Drug Administration (FDA) and the European Medicine Agency (EMA) for use in humans [5]. PLGA can be manufactured in different ratio proportions and molecular weights to encapsulate hydrophobic and hydrophilic molecules. Moreover, it is useful as a carrier for micro and macromolecules. Because of these advantages, PLGA has been chosen for numerous systems in biomedical applications, including cancer diagnosis and treatment, vaccination, inflammation treatment, or neurodegenerative and cardiovascular diseases [6,7,8,9,10,11,12]. However, PLGA-NPs are easily recognized by the reticuloendothelial system (RES), and consequently, it aids their fast elimination from the bloodstream. An alternative to avoid this is to coat these systems with antibodies, chitosan, poloxamer, or polyethylene glycol (PEG). This can increase their half-life in the bloodstream [13,14] and help their transport through membranes [15]. In addition, PEG is useful to stabilize NPs in aqueous media, avoiding aggregation and increasing their solubility [16,17,18].

Drying, by converting suspensions or liquids into dried products, is a technique commonly used for improving the stability of pharmaceutical and biotherapeutic products [19,20,21,22,23]. A drying process to remove the water is highly recommended to maintain NPs’ respective physicochemical properties, efficacy, and long-term stability and to avoid instability that is caused by aqueous media [24].

Techniques such as spray drying [25,26,27] and freeze-drying (lyophilization) [28,29,30] have been described in the literature for the conversion of colloidal suspensions into dried products. The spray drying process was considered as a dehydration method, but at the same time, it has increasingly attracted research interest for the design of pharmaceutical carriers encapsulating drugs [31,32]. Its use has been demonstrated in the food [33], chemical, and pharmaceutical industries, being one of the candidate methods to offer an alternative to lyophilization in the future [19,34,35,36,37]. The advantages of this technique over conventional freeze-drying are the shorter process cycle time, scalability, affordability, reproducibility, and the fact that it works at atmospheric pressure. Moreover, the production cost is typically lower compared to other drying technologies [32].

Significant efforts have been made over the last few decades to develop new drug delivery systems that improve ocular drug administration. The use of colloidal delivery systems, such as NPs, is considered as a strategy that can enhance the ocular bioavailability of topically administered drugs, reducing administration frequency, sustaining the release, and promoting drug targeting to specific sites [38,39,40,41,42,43,44,45].

In recent years, studies of pioglitazone (PGZ) as a potential therapeutic have gone far beyond its primary use as an antidiabetic drug. This drug is an agonist of the peroxisome proliferator-activated receptor γ (PPARγ) which is a ligand-activated nuclear transcription factor that modulates the expression of genes involved in the physiological functions, including glucose homeostasis, lipid metabolism, and inflammatory processes. Additionally, PGZ has been reported to have different therapeutic activities, including its regulatory role on inflammatory markers, such as IL-6, IL-17, IFN-γ, TNF-α. In addition, its use in autoimmune diseases, rheumatoid arthritis, cardiovascular diseases, skin inflammatory diseases, Alzheimer’s [46,47,48,49,50], and for the treatment of ocular disorders [51,52,53] suggest that this drug could be an effective candidate for the treatment of different inflammatory ocular conditions [51].

Previous studies have shown that PGZ was encapsulated into PLGA-PEG-NPs, suggesting its suitability as a pharmaceutical formulation that overcomes the poor solubility of PGZ in an aqueous phase [54] and targets specific tissues [46,55,56]. Moreover, this system showed good corneal and scleral permeation and sustained release, indicating these systems could be useful for drug delivery [52]. However, in order to preserve and increase the long-term stability of PGZ-NPs for future use as a drug medicine, the water must be removed and these systems transformed into a dry powder.

The purpose of this study was to optimize and stabilize the PGZ-NPs of PLGA-PEG by the spray drying technique, improving its physicochemical characteristics, biopharmaceutical behavior, and long-term stability. Moreover, release profile, in vivo and ex vivo ocular permeation studies were conducted. In addition, corneal analysis by synchrotron X-ray scattering was performed for future use of these systems in an ocular inflammatory animal model.

## 2. Materials and Methods

### 2.1. Materials

Diblock copolymer PLGA-PEG 5% (50:50) Resomer® was obtained from Evonik Corporation (Birmingham, AL, USA). The PGZ was obtained from Capot Chemical (Hangzhou, China). Acetone, dimethylsulfoxide (DMSO) and polyvinyl alcohol Mw 30,000–70,000 Da, 87–90% hydrolyzed (PVA) were purchased from Sigma-Aldrich (Darmstadt, Germany). Transcutol^®^ P pharma quality was kindly supplied by Gattefossé España S.A. (Barcelona, Spain). Milli-Q water (Millipore Sigma, Burlington, MA, USA) was used for all the experiments and the reagents were of analytical grade.

### 2.2. Experimental Strategy

i. Develop and select the most suitable PGZ-PLGA-PEG NP colloidal system based on physicochemical properties.
ii. Dry the selected nanosystem with spray dryer Büchi Nano B-90 under different conditions and select the most suitable formulation in terms of physicochemical properties, in vitro release, and ex vivo corneal and scleral permeation in comparison to the colloidal system without drying.
iii. Bioavailability study of PGZ from the NPs in rabbit eyes with both selected formulations: suspension freshly prepared (Table 1–formulation 8) and reconstituted suspension after drying (Table 2–Spray 2).
iv. Small-angle X-ray scattering (SAXS) analysis in the corneal tissue.

### 2.3. Development and Preparation of PGZ-NPs

The experimental strategy used in this work for the preparation of PGZ-NPs by the solvent displacement technique was a factorial design, which consists of simultaneously modifying the different variables within the experimental domain. Thus, the number of experiments in a complete factorial design is equal to the number of possible combinations between the levels (*n*) of each variable (X): *n*^x^. A screening factorial design 2^3^ was planned to develop the PGZ-NPs and to analyze the effect of independent variables (pH, PLGA-PEG and PVA concentrations) together and in combination with the dependent variables (average particle size (Z_av_), polydispersity index (PI), zeta potential (ZP), and encapsulation efficiency (EE)). According to the composite design matrix generated by Statgraphics Centurion 18 software (Statgraphics Technologies Inc., The Plains, VA, USA), a total of 8 experiments are summarized in Table 1.

PGZ-NPs were prepared using the nanoprecipitation technique firstly described by Fessi et al., 1989 [57]. Previously the PGZ (1 mg/mL) was solubilized in 0.5 mL of DMSO, then mixed with the PLGA-PEG dissolved in 5 mL of acetone. This organic phase was added dropwise under stirring (700 rpm) into 10 mL of an aqueous solution of PVA adjusted to the desired pH. Then, acetone was evaporated under reduced pressure in a rotary evaporator Büchi B-480 (Büchi, Fawil, Switzerland). The PGZ-NP suspensions were stored until spray drying.

### 2.4. Spray Drying of PGZ-NPs

Nano Spray Dryer B-90 (Büchi Labortechnik AG, Fawil, Swiitzerland) was used to improve the long-term stability of the PGZ-NPs. This instrument carries out the spray drying process using vibrating mesh technology to generate the droplets [58] and the dried particles are collected by an electrostatic accumulator (collector). This technology minimizes product loss and provides high yields up to 90% [31,59].

Four different dry particles were prepared from selected PGZ-NP formulations. The instrument conditions tested were spray nozzle (5.5 and 7.0 µm) and outlet temperatures around 25 and 30 °C. The set up for Spray 1 was 5.5 µm and 25 °C; 7.0 µm and 25 °C for Spray 2; 5.5 µm and 30 °C for Spray 3; and 7.0 µm and 30 °C for the Spray 4 formulation. The air flow rate was set to 140–150 L/min and the spray rate was fixed at 75% for all conditions. The PGZ-NP solution was sprayed, and particles were collected dried from the electrostatic particle chamber using a powder scraper. After that, samples were maintained in a desiccator until further analysis.

### 2.5. Physicochemical Characterization

The Z_av_ and PI measurements of PGZ-NPs were performed by Dynamic Light Scattering (DLS) using a Zetasizer Nano ZS (Malvern Instruments Ltd., Malvern, UK) at 25 °C using disposable quartz cells, with prior dilution in Milli-Q water (1:20 *v*/*v*) to determine hydrodynamic radii of particles in water. ZP was determined by electrophoresis laser-Doppler using the same instrument with prior dilution in Milli-Q water (1:10 *v*/*v*). All the experiments were carried out in triplicate.

To determine the EE percentage of PGZ in NPs, an indirect method was used of dilution of 1:10 (*v*/*v*) in water and filtration/centrifugation at 14,000 rpm for 20 min. The amount of non-entrapped PGZ found in the supernatant was determined by high-performance liquid chromatography (HPLC) using a previous validated analytical method [46] and calculated according to the following Equation (1):

(1) $$EE\;\left(\%\right)=\frac{Total\;amount\;of\;PGZ-Free\;PGZ}{Total\;amount\;of\;PGZ}\;\times \;100$$

### 2.6. Water Content

Water content was determined using a selective electrochemical sensor of phosphorus pentoxide (P_2_O_5_) by means of thermo-coulometric analysis with the Berghof easyH_2_O analyzer (DKSH, Zurich, Switzerland). Basically, after instrument calibration, 10 mg of dried sample was heated to 150 °C in a closed chamber for 10 min, the evaporated water was conveyed to the sensor in a stream of carrier gas, and the free surface water, capillary water, and bonded water were calculated through Faraday‒s first law of electrolysis. The moisture is attracted to the P_2_O_5_ coating, which is very hygroscopic and migrates to the electrodes (platinum wires) where it is electrolyzed. Oxygen is formed at the positive electrode and hydrogen at the negative electrode. The electrolysis current, according to Faraday’s law, is directly proportional to the amount of water [60].

### 2.7. Microscopy Analysis (TEM and SEM)

PGZ-NP dispersions were analyzed before and after the spray drying process by transmission electronic microscopy (TEM) and scanning electron microscopy (SEM), respectively. TEM analysis was carried out by negative stain technique. Samples were placed on the grid surface after a dilution in water (1:4 *v*/*v*) mounted on a grid and negative stained with a 2% (*v*/*v*) uranyl acetate solution. After 6 h at room temperature, the samples were examined by TEM on a JEOL JEM-1010 (JEOL USA, Peabody, MA, USA).

After the spray drying process, the PGZ-NPs were analyzed by SEM. For that purpose, a small quantity of PGZ-NPs was coated with carbon as a conductor agent, and the structure was examined using a JEOL J-7100F (JEOL USA, Peabody, MA, USA).

### 2.8. Interactions Studies: DSC, X-ray Spectroscopy, and FTIR

To determine the physical state of the PGZ-NPs and their interactions with any compound of the formulation, studies by Differential Scanning Calorimetry (DSC), X-ray spectroscopy, and Fourier Transform Infrared (FTIR) were performed before and after the spray drying process.

DSC analysis was performed using a DSC 823e System (Mettler Toledo, Barcelona, Spain). The temperature was calibrated by the melting transition point of indium (purity ≥ 99.95%; Fluka, Switzerland) prior to sample analysis. The DSC measurements used a heating ramp from 30 to 240 °C at 10 °C/min in a nitrogen atmosphere. Data were evaluated using the Mettler STARe V 9.01 dB software (Mettler Toledo, Barcelona, Spain).

To determine the state—crystalline or amorphous—of the samples, X-ray spectral measurements were performed using Siemens D500 system (Karlsruher, Germany). X-ray powder diffractograms were recorded using a Cu Kα2 radiation (45 kV, 40 mA, λ = 1.544 Å) in the range (2θ) from 2° to 60° with a step size of 0.026° and the measuring time was 200 s per step.

FTIR spectra were achieved using a Thermo Scientific Nicolet iZ10 with an ATR diamond and DTGS detector (Thermo Fischer Scientific, Waltham, MA, USA). The scanning range used was 500–4000 cm^−1^.

### 2.9. Release Profile of PGZ-NPs

Drug release studies of PGZ from the NPs before and after the spray drying process were performed using the Franz diffusion cell technique, which is based on the direct dispersion of the colloidal suspension in the dialysis medium accomplishing sink conditions. A dialysis membrane (MWCO 12,000–14,000 Da Dialysis Tubing Visking, Medicell International Ltd., London, UK) was used and hydrated for 24 h before being mounted in the Franz diffusion cell. The temperature of the medium and speed of the paddle were adjusted to 37 ± 0.5 °C and 100 rpm, respectively. The PGZ-NP formulations were compared with respect to the free drug at the same concentration. The free drug was dissolved in DMSO and phosphate buffer solution (PBS) at pH 7.4 (60:40 *v*/*v*). This solution 60:40 *v*/*v* was also the release media (RM). A volume of 300 μL of PGZ-NPs or free drug was placed in the donor compartment, and the receptor compartment was filled with RM. At predetermined time intervals, 300 μL was withdrawn from the receptor compartment and replaced with an equivalent volume of RM. The concentration of PGZ released was measured by liquid chromatography [46]. Values are reported as the mean ± SD of six replicates. The content of PGZ in the RM at each time point was evaluated and data were adjusted to the most common kinetic models. Akaike’s information criterion (AIC) and determination coefficient (*r^2^*) were determined for each model [61].

### 2.10. Corneal and Scleral Permeation Studies

In order to continue with the following experiments, based on the physicochemical characterization and biopharmaceutical studies of the PGZ-NPs (Section 3), the Spray 2 formulation after the drying process was selected (see Table 1).

Franz diffusion cells were used in order to study permeation through corneal and scleral tissue. The ocular tissue was obtained from male New Zealand rabbits weighing (2.5–3 kg, *n* = 3) under veterinary supervision, and according to the Ethics Committee of Animal Experimentation from the University of Barcelona (CEEA-UB). The rabbits were anesthetized with an intramuscular administration of ketamine HCl (3 mg/kg) and xylazine (2.5 mg/kg). Once sedated, they were euthanized by an overdose of sodium pentobarbital (100 mg/kg), administered through a marginal ear vein under deep anesthesia. The ocular tissues (cornea and sclera) were excised and immediately transported to the laboratory in artificial tear solution. The tissues were placed in Franz diffusion cells between the donor and receptor compartment with a permeation area of 0.64 cm^2^. The receptor compartment was filled with freshly prepared solution Transcutol/water (60/40 *v*/*v*). A sample volume of 0.2 mL from PGZ-NPs and the Spray 2 was added in the donor compartment. The drug release from the receptor compartment was studied for 6 h by removing 0.2 mL of sample and replacing it with fresh solution of Transcutol/water. This compartment was kept at 32 ± 0.5 and 37 ± 0.5 °C for corneal and scleral permeation, respectively. The cumulative PGZ amount permeated was calculated, at each time point, from concentration of PGZ in the receiving medium and plotted as function time (hour). HPLC was used to analyze the samples [46].

### 2.11. Ocular Permeation Parameters

After a 6 h ex vivo experiment, the cornea and sclera were cleaned using a 0.05% solution of sodium lauryl sulfate and washed with distilled water, then weighed and the PGZ extracted with methanol under sonication for 30 min using an ultrasound bath. PGZ levels were quantified by HPLC and expressed as (µg.g^−1^.cm^−2^) of cornea or sclera extracted (*Q_ext_*) and retained (*Q_ret_*) through the tissues. The permeated (*Q_t_*) amount was expressed as µg.cm^−2^. In addition, the permeability coefficient (*Kp*) (cm·h^−1^), latency period (*T_L_*), and steady-state flux (J) for the scleral tissue were calculated by plotting the cumulative PGZ permeating versus time, determining x-intercept by linear regression analysis.

### 2.12. In Vivo Study

In vivo study was carried out in order to investigate ocular bioavailability and disposition of PGZ from NPs before and after the spray drying process. New Zealand rabbits weighing (2.5–3 kg, *n* = 3) were used in accordance with the Ethics Committee of Animal Experimentation from the University of Barcelona (CEEA-UB). Rabbits were euthanized after 10 d of topical administration with PGZ-NPs and the Spray 2 formulation (0.05 mL/eye/day). The ocular tissues were isolated from the eyes and kept at −80 °C until quantification by liquid chromatography–mass spectrometry (LC-MS) [62]. The values are described as the average ± SD (*n* = 3/group).

### 2.13. Small-Angle X-ray Scattering (SAXS) Data Collection and Analysis

After the in vivo study, corneal tissue from rabbits treated with saline solution 0.09% (negative control), PGZ-NPs freshly prepared, and the Spray 2 formulation, were dissected and maintained in a formaldehyde 4% solution until the analysis by SAXS on beamline BL11-NCD-SWEET at the ALBA Synchrotron Light Source (Barcelona, Spain). A thin strip of tissue, measuring approximately ~1 mm wide, was dissected from the center of each cornea from the anterior to posterior direction covering from limbus-to-limbus. The cuts were wrapped in plastic membrane in order to prevent dehydration during data collection. The samples were mounted in a Perspex-Mylar holder and positioned perpendicular to the beam and on motorized stages that permitted performing automated raster scans. Each pattern was generated from an X-ray beam with an energy of 12.4 keV and a sample to detector distance of 7 m. AgBh (with a standard periodicity of 58.38 Å) was used in order to perform spatial calibration of data using PyFAI (Grenoble, France). SAXS patterns were obtained at 25 µm intervals in a vertical direction throughout the thickness of the cornea center, scanning from epithelium to endothelium, with an exposure time of 4 s. A total of 35 data points were collected from each cornea, and for each group, two corneas were examined. The analysis of the images was carried out using the SAXS4COLL software (Available online: https://bio.tools/saxs4coll Acceded date: 12 February 2021. N/A version, Cardiff, UK) [63] which was developed for the analysis of fibrous collagen-based tissues.

### 2.14. Stability Studies

Long-term stability of PGZ-NPs and the Spray 2 formulation (once reconstituted) was carried out for 6 months at 4 °C. Turbiscan Formulaction Lab® was used to analyze the backscattering profile of the samples.

### 2.15. Statistical Analysis of Synchrotron Data

Data were analyzed using the GraphPad Prism version 8 software package (GraphPad Software Inc, San Diego, CA, USA). Differences between treatment groups and the control were statistically evaluated using ANOVA. The average of fibrillar diameter and interfibrillar spacing of collagen was calculated out of the 35 points that were collected from each cornea in the different groups. Differences were considered as statistically significant when the *p*-value was less than 0.05. All the data are presented as the average ±SD.

## 3. Results

### 3.1. Development and Characterization

**Figure 1:**
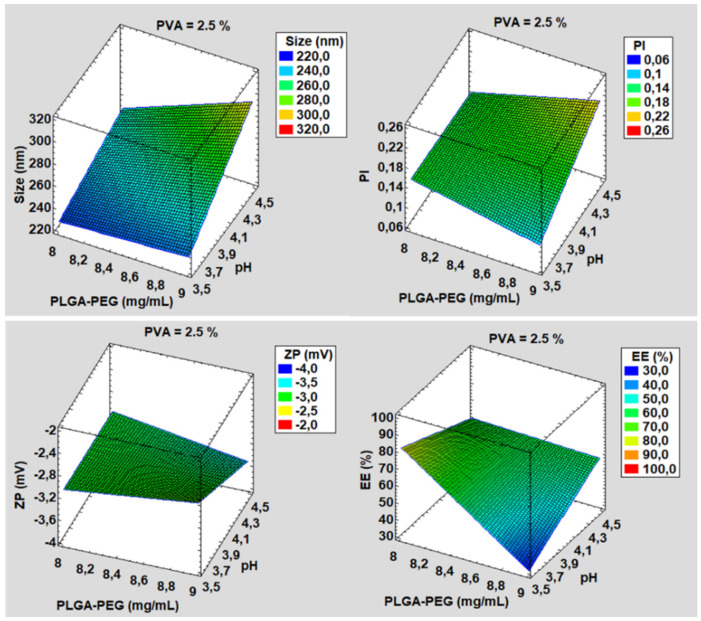
Surface response of PGZ-NPs developed by factorial design before the spray drying process. PVA=2.5%. pH and PLGA-PEG concentration influence on the size, PI, ZP and EE % of NPs.

The PGZ-NPs were developed from a factorial design 2^3^ (Figure 1) and prepared using a solvent displacement technique [57]. Eight formulations were developed and their physicochemical parameters with respect to size, PI, ZP and % EE were determined to select the most suitable formulation according to those parameters and to be dried by spray drying technology (Table 1). The mean particle size ranged from 223.90 ± 1.60 to 302.81 ± 1.94 nm, PD values were below 0.23, ZP were around –3.5 mV, and EE values ranged from 35.74 to 90.12%. The formulation number 8 of PGZ-NPs was selected based on its physicochemical characteristics, mainly the percentage of EE, which was significantly higher in comparison to other formulations.

After that, the experiment using the Nano Spray Dryer B-90 was carried out with this particular formulation. From here, four PGZ-NPs were obtained with different spray conditions (see Section 2.4). Parameters such as size, PI, ZP and EE % were determined (Table 2). The particles showed a slight decrease in the surface charge after spraying as well as a slight increase in mean particle size.

**Figure 2:**
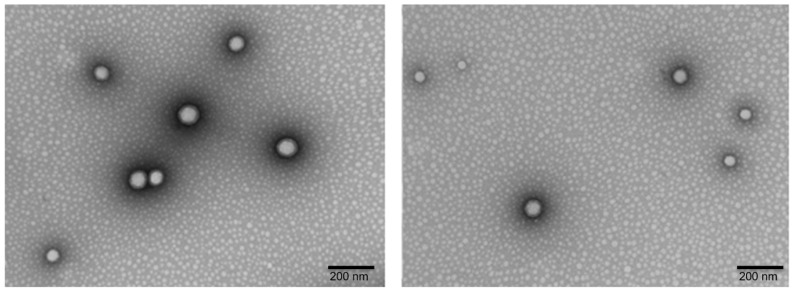
TEM images of PGZ-NPs in suspension before spray drying.

**Figure 3:**
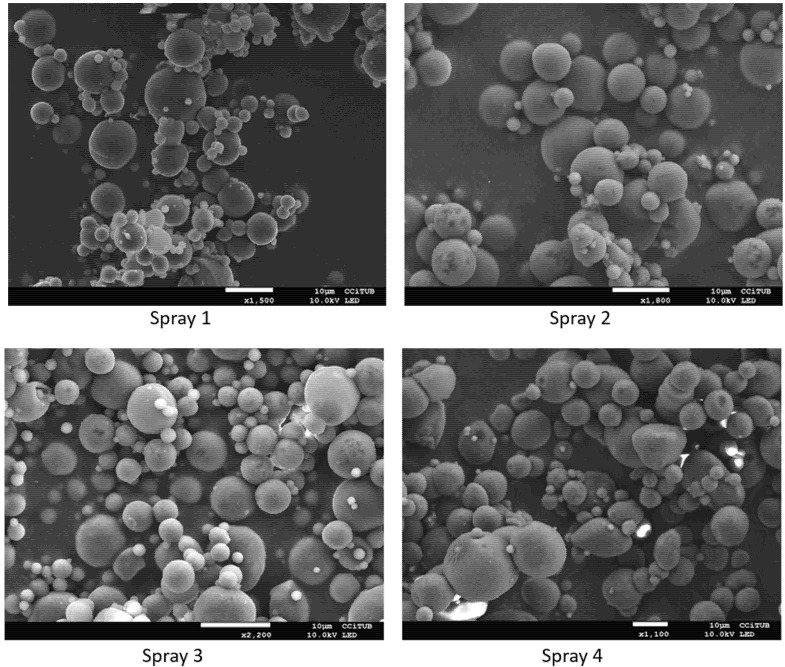
SEM images of PGZ-NPs after spray drying.

Moreover, size and surface morphology of the selected PGZ-NPs were determined by TEM (Figure 2), evidencing that PGZ-NPs were morphologically spherical and presented low dispersion. In addition, after the spray drying process, TEM (Figure 3) and SEM (Supplementary Materials, Figure S1) determinations were performed in order to ensure the drying process did not change the morphology of the particles.

SEM images of NP-based dried solids showed a high polydispersity of the aggregates present in the powder, from nanometers to micrometers in diameter. This is the usual phenomenon when using the spray drying technology to dry this type of suspension [64]. After the resuspension of the NP-based dry solids in water (Figure S1), it was possible to observe an acceptable polydispersity (PI 0.24) that was correlated with DLS measurements (Table 2).

### 3.2. Water Content

The water content remaining in the nanoparticle-based dry powder after the spray drying process was investigated. Spray 1, 2, 3, and 4 formulations showed water contents of 2.62, 2.15, 3.24, and 3.15% wet basis, respectively.

### 3.3. Interaction Studies for PGZ-NPs and Their Components Using the Nanoparticle-Based Dry Powder

**Figure 4:**
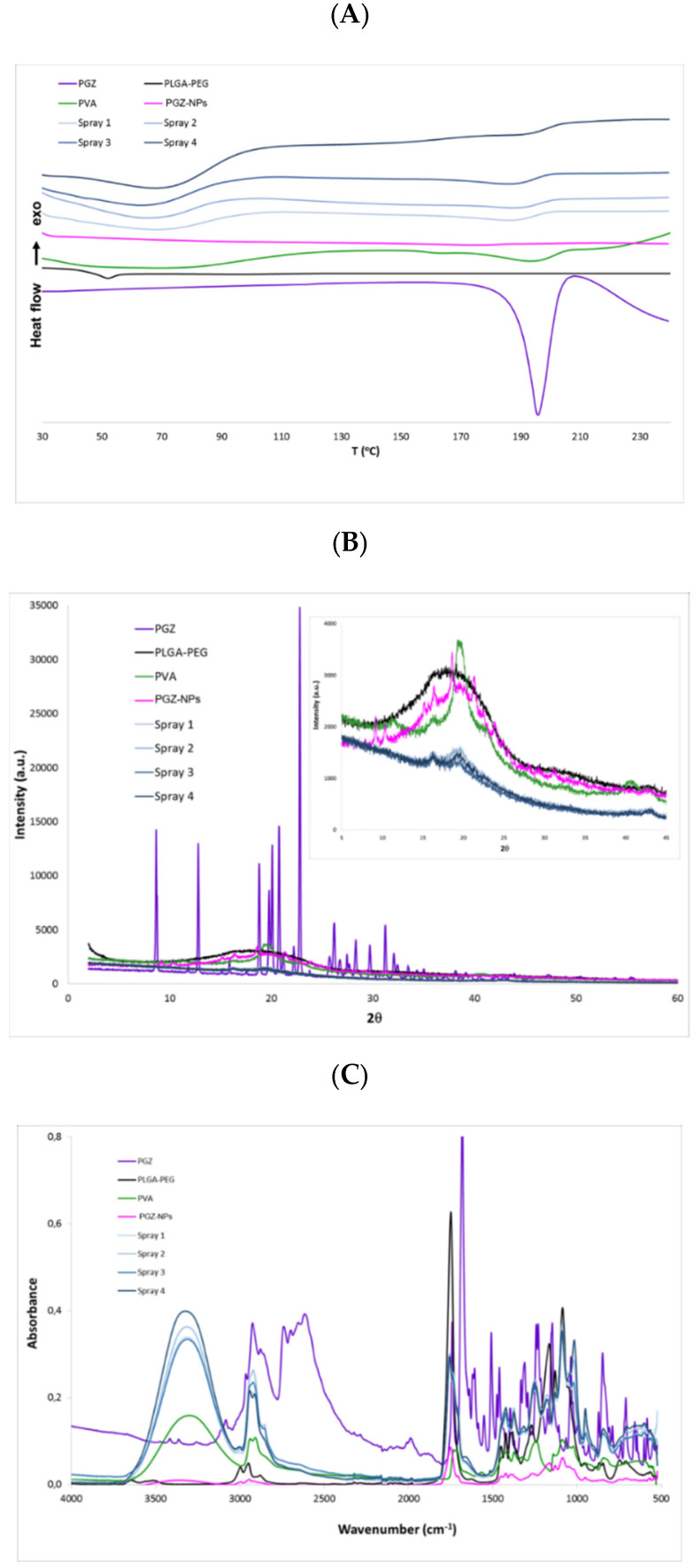
Interaction studies for PGZ-NPs. (**A**) Differential Scanning Calorimetry, (**B**) X-ray diffraction patterns, (**C**) FTIR spectra.

A factor that greatly affects the release of the drug in in vitro and in vivo studies is the physical state of the drug within the NPs. Therefore, PGZ was characterized by DSC, X-ray diffraction, and FITR (Figure 4A, Figure 4B and Figure 4C, respectively). During DSC analysis the produced thermogram showed an endothermic accident that corresponded to the fusion of the drug with a maximum temperature of 195.90 °C and an enthalpy of 140.33 J/g. In addition, individual thermograms of each component of the study were obtained: PVA, PLGA-PEG, PGZ-NPs before and after the spray drying process (Figure 4A). The PVA showed an endothermic event corresponding to the melting process with a maximum at 193.55 °C and an enthalpy of 5.32 J/g. The PLGA-PEG glass transition (Tg) temperature was around 50 °C. However, when the method of removing water from the suspension of the NPs was carried out using the spray drying technique, the DSC analysis did not provide information of interest. The signal from the surfactant (PVA) overshadowed the other signals, since PVA is not removed when using this drying process. For this reason, in order to assess the effects of this process on the particles, a size control (Z_av_), sample homogeneity (PI) and EE were carried out after reconstitution of the sample formulation.

The diffraction pattern of the drug indicated a crystalline structure unlike the polymer (Figure 4B). The PVA, PGZ-NPs, and each NP formulation after the drying process (Spray 1, Spray 2, Spray 3, Spray 4) showed an amorphous structure. PGZ-NPs before the the drying process showed a profile similar to the PLGA-PEG, with small and attenuated peaks that corresponded to those that appeared in the drug diffraction pattern (Figure 4B).

The formation of new bonds was not observed. Related to the dry samples by spray drying, the absorption peaks were very similar to each other and corresponded mainly to the surfactant (PVA) (Figure 4C).

### 3.4. Release Profile

**Figure 5:**
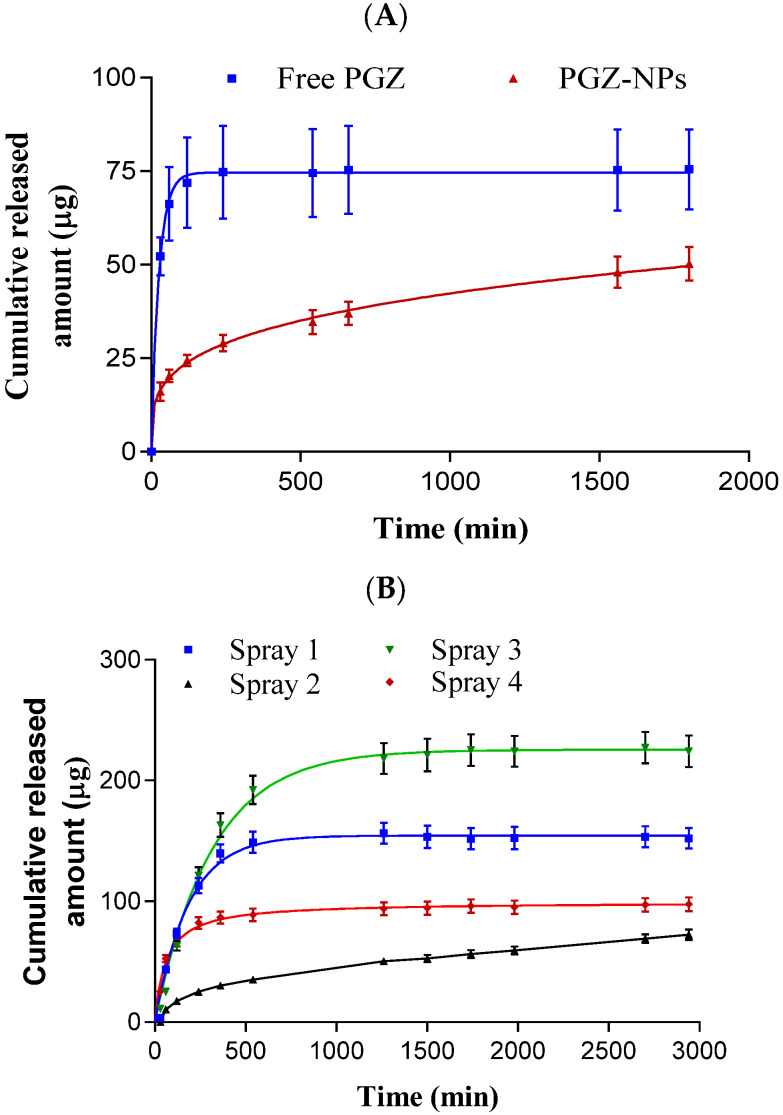
Release profile of PGZ-NPs before (**A**) and after (**B**) the spray drying process (*n* = 6).

The release of PGZ from PGZ-NPs and from the nanoparticle-based dry powder coming from the spray drying process was evaluated before (Figure 5A) and after resuspension in water (Figure 5B). Different kinetic models were applied to evaluate the best kinetic profile for both cases (Supplementary Materials, Table S1). PGZ-NPs showed an AIC value of 13.20 and *r^2^* of 0.99, adjusting well to the Korsmeyer–Peppas kinetic profile. After the drying process of the four formulations, the Spray 2 formulation was selected based on the similar kinetic profile and parameters before drying. The Spray 2 formulation showed values for AIC and *r^2^* of 46.02 and 0.99, respectively. Moreover, it adjusted to Korsmeyer–Peppas kinetic profile.

### 3.5. Ex Vivo Permeation Studies

**Figure 6:**
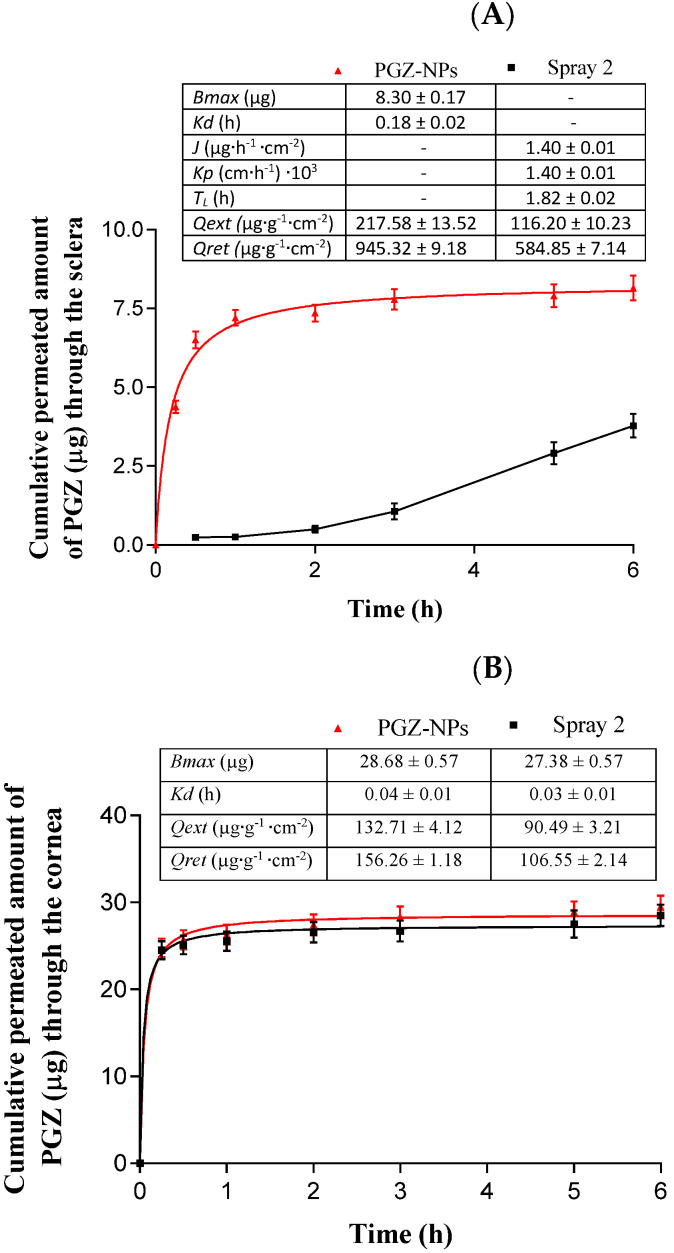
Permeation studies of PGZ-NPs and the Spray 2 formulation in the scleral tissue (**A**) and corneal tissue (**B**). The values are expressed by mean ± SD (*n* = 3).

Regarding the scleral tissue, PGZ-NPs and the Spray 2 formulation fitted to hyperbola and first order profiles, respectively (Figure 6A). PGZ-NPs showed a maximum quantity released (*Bmax*) of 8.30 µg and a retained quantity (*Qret*) in the tissue of 945.32 µg.g^−1^.cm^−2^. The profile for the Spray 2 formulation was a conventional infinite dose, showing a flow penetration (*J*) of 1.40 µg.h^−1^.cm^−2^ and a latency period (*T_L_*) of 1.82 h. Moreover, a retained amount (*Qret*) of 584.85 µg.g^−1^.cm^−2^ was lower than PGZ-NPs.

For the corneal tissue, the permeation profiles for both formulations were similar (Figure 6B). They showed a hyperbola profile with a *Bmax* of 28.68 µg for PGZ-NPs and 27.38 µg for the Spray 2 formulation. The *Qret* values for PGZ from NPs and the Spray 2 formulation were 156.26 and 106.55 µg.g^−1^.cm^−2^, respectively.

Permeated amounts of PGZ from NPs and the Spray 2 formulation at 3 h in the scleral tissue were 12.96 and 1.56 µg.cm^−2^, respectively. In the case of the corneal tissue, at the same time they were 39.84 and 39.72 µg.cm^−2^, respectively.

### 3.6. In Vivo Studies and X-ray Synchrotron Corneal Analysis

**Figure 7:**
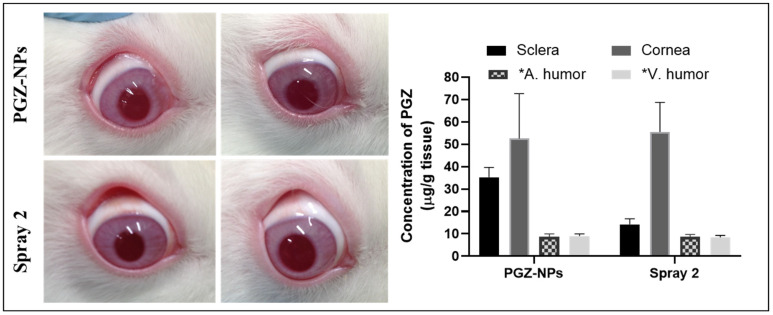
Bioavailability study. Levels of PGZ after 10 d of administration of PGZ-NPs and the Spray 2 formulation. * Vitreous Humor and Aqueous Humor: data are expressed as µg/mL. The values are expressed by mean ± SD (*n* = 3).

Freshly prepared PGZ-NPs and Spray 2 formulation were administrated in rabbit eyes for 10 d and after that, the eyes were isolated, and the different ocular tissues were separated (Figure 7). PGZ from NPs was detected in the cornea, sclera, vitreous and aqueous humor at concentrations of 52.57 ± 20.11 µg/g, 35.31 ± 4.32 µg/g, 9.06 ± 0.90 µg/mL and 8.76 ± 1.20 µg/mL, respectively. PGZ from the Spray 2 formulation was detected in the same tissues, with concentrations in cornea, vitreous and aqueous humor of 55.47 ± 13.26 µg/g, 8.46 ± 0.82 µg/mL, and 8.70 ± 1.00 µg/mL, respectively. A lesser concentration, 14.26 µg/g, was found in scleral tissue.

**Figure 8:**
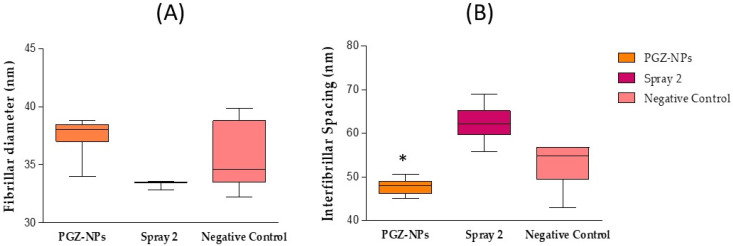
SAXS corneal synchrotron analysis. Collagen fibrillar diameter (**A**) and collagen interfibrillar spacing (**B**) (*n* = 2) (* *p* < 0.05).

After the in vivo study, the corneal tissue was also evaluated by X-ray synchrotron analysis. The data obtained are the mean of 35 points in each cornea in the treatment groups. Both corneas for each treatment group were taken from the same animal in order to discard genetic factors in the analysis of data. Figure 8A,B represent the obtained values of fibrillar diameter and interfibrillar spacing of the collagen for each treatment, in box and whisker plots, respectively.

The cornea treated with PGZ-NPs showed an average value for collagen fibrillar diameter of 37.7 ± 0.8 nm. For treatment with the Spray 2 formulation the average value was 33.5 ± 0.1 nm, and 37.6 ± 1.9 nm in the untreated cornea (negative control). The interfibrillar spacing was 47.7 ± 1.5 nm for PGZ-NPs treatment, 62.2 ± 0.3 nm for the Spray 2 formulation treatment, and 52.8 ± 4.4 for the untreated cornea (Figure 8). The parametric, as well as the non-parametric ANOVA tests, revealed no significant differences in the fibrillar diameter between untreated and PGZ-NP treatment (*p* > 0.05). The same test was applied to the interfibrillar spacing and showed significant differences between PGZ-NPs and the Spray 2 formulation. The corneal treatment with the Spray 2 formulation showed a decrease in the fibrillar diameter with respect to the untreated eye and the treated eye with PGZ-NPs not previously dried.

Although no explanation for this behavior was deduced in this study, we can conclude that the cornea was not damaged by the treatment with PGZ-NPs or the Spray 2 formulation since the values found for interfibrillar spacing and fibrillar diameter showed that the cornea did not lose its properties.

### 3.7. Stability Studies

Long time stability was determined using Turbiscan Formulaction Lab ® (Formulaction, Toulouse, France) for PGZ-NPs and the Spray 2 formulation over 6 months at 4 °C. Figure 8 shows that both formulations were stable over this time since fluctuation in backscattering signals was lower than 10% [40,65].

## 4. Discussion

PGZ-NPs were successfully developed by factorial design 2^3^ and synthetized with high-performance using the nanoprecipitation method [57]. Based on the physicochemical characteristics of these systems (see Table 1), formulation number 8 was selected. These NPs showed an adequate size (247.22 nm), low PI (0.17), and a high percentage of EE (around 90%). PLGA-PEG is a very useful polymer to encapsulate different components, such as lipophilic and hydrophilic drugs and proteins [18,66,67]. The selected PGZ-NPs were stabilized under four different conditions of drying using Nano Spray Dryer B-90. This technique was applied to encapsulate drugs, preserving the included compound and also improving the % EE [31]. TEM and SEM images showed spherical and monodisperse particles before and after the spray drying process (Figure 2, Figure S1 and Figure 3, respectively).

Regarding the interaction’s studies, DSC analysis showed a decrease in the Tg when the PEG was linked to the PLGA [68]. The decrease value after the incorporation of the PEG was due to a plasticizing effect based on the reduction of the attractive forces between the polymer chains [68]. The thermogram of the PGZ-NPs did not show the drug fusion, which indicated that the PGZ was inside the particles in the form of a molecular dispersion or in a solid solution (Figure 4A) [68]. Nevertheless, in the DSC analysis of the samples (Spray 1, Spray 2, Spray 3, and Spray 4) it was not possible to observe the effects of the drying process on the Tg, or the effect that the drying process could have had on the drug. This was due to the fact that the PVA was not removed in the drying process, and its signal appeared to overshadow the other signals. For that reason, X-ray and FTIR were carried out to corroborate these data.

The X-ray diffraction profiles showed that PGZ had a crystalline structure unlike the polymer, the surfactant, the PGZ-NPs, and spray formulations, which showed an amorphous structure. These results indicated that when the PGZ was loaded in the form of NPs, it showed a similar polymer profile (Figure 4B). These results agreed with previous studies [30,40,52,69,70]. The different drying conditions were not reflected in the X-ray diffraction profiles since they were similar, basically corresponding to the PVA profile as seen in Figure 4B. These results agreed with those obtained by DSC.

Regarding FTIR analysis, the formation of new covalent bonds was not observed (Figure 4C). These results were in accordance with other authors [30,45]. Related to the drying formulation, the absorption peaks were very similar and corresponded mainly to the surfactant, as in the previous cases.

Furthermore, for the release profile, PGZ-NPs before drying showed an AIC of 13.20 and sustained release up to 30 h (Figure 5A). After drying the four spray formulations, the most similar release profile with respect to the previous one was the Spray 2 formulation (Figure 5B). PGZ-NPs before and after drying showed a sustained and slow profile, and both fitted to the Korsmeyer–Peppas kinetic model (Supplementary Materials Table S1). These results were in accordance with previous studies using similar polymers [68,71].

Parameters for ex vivo permeation in corneal and scleral tissue were determined (Figure 6). As shown in Figure 6A, PGZ-NPs before drying showed higher values for *Qret* and *Qt* (946.32 µg.g^−1^.cm^−2^ and 12.96 µg.cm^−2^, respectively). However, at 6 h when the flow was in steady state, the Spray 2 formulation increased the *Qt* value to almost double with respect to PGZ-NPs. These results were in accordance with previous data [40,45,52,70] suggesting that these formulations could be used for the posterior pole. Nevertheless, for the corneal tissue (Figure 6B), the *Qret* was higher before drying, while the *Qt* values at 3 h for both formulations were very similar, showing values around 40 µg.cm^−2^ and a *Kd* around 0.04 h, indicating that both formulations could have similar therapeutic effects in the anterior pole of the eye.

After 10 d of topical application of PGZ-NPs and the Spray 2 formulation (0.05 mL/eye/day), PGZ was detected in different ocular tissues, including cornea, sclera, aqueous, and vitreous humors (Figure 7). Similar values for PGZ were found in the corneal tissue before and after the drying process, indicating that the spray drying process did not compromise the PGZ permeation through the tissue. However, for the scleral tissue, a decrease in the PGZ value was observed when using the aqueous suspension of NPs-based dried powder. Nevertheless, it did not compromise its effectiveness. These data correlated with those obtained in ex vivo corneal and scleral permeation studies [40,52].

The corneal tissue was analyzed by SAXS with synchrotron light to observe if, after in vivo application of PGZ-NPs and the Spray 2 formulation, the collagen had been damaged. Given the variability in the data of the fibrillar diameter and the interfibrillar spacing of collagen between treated and untreated eyes, it was possible to observe that PGZ-NPs before and after the spray drying process did not produce significant changes in the physical properties of collagen in the cornea with respect to the negative control (Figure 8) and hence, the corneal ultrastructure was not damaged post-treatment.

**Figure 9:**
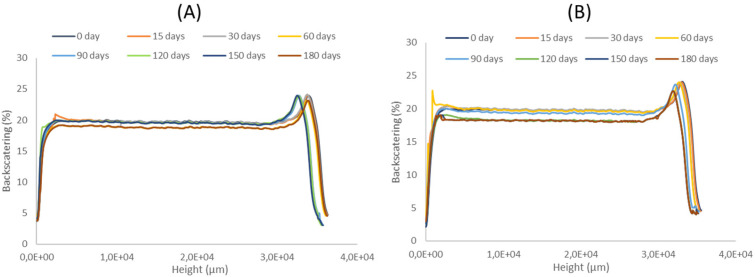
Backscattering profiles of colloidal suspensions at 4 °C. (**A**) PGZ-NPs and (**B**) Spray 2 formulation.

Regarding the stability of these systems, PGZ-NPs and the Spray 2 formulation were studied for 6 months at 4 °C (Figure 9). Both formulations were shown to be stable under those conditions and did not present a significant variation in the percentage of backscattering. These results were in accordance with other studies in which PLGA-NPs were stable for a quite long times [40,65,72].

## 5. Conclusions

A comparative study was performed using PGZ-NPs before and after stabilization by the Nano Spray Dryer B-90, including in vitro, ex vivo, in vivo, and stability studies. This technique demonstrated that, the structure was physically and morphologically maintained after stabilization. Moreover, the spray drying process did not affect the corneal bioavailability. Furthermore, the collagen structure in the corneal tissue remained intact after the treatment of rabbit eyes with PGZ-loaded PLGA-PEG NPs. The spray drying technique can be an alternative to stabilize PGZ-NPs for future therapeutic use.

## Figures and Tables

**Table 1:** PGZ-NPs developed by a factorial design 2^3^.

Formulation	PLGA-PEG (mg/mL)	pH	PVA (%)	Size (nm)	PI	ZP (mV)	EE (%)
1	8.0	3.5	2.0	223.90 ± 1.60	0.09 ± 0.00	−2.47 ± 0.08	39.45 ± 2.12
2	9.0	3.5	2.0	231.91 ± 0.77	0.08 ± 0.01	−3.30 ± 0.30	48.95 ± 1.34
3	8.0	4.5	2.0	242.32 ± 2.85	0.16 ± 0.03	−2.54 ± 0.43	79.23 ± 3.21
4	8.0	3.5	2.5	251.42 ± 0.66	0.20 ± 0.00	−3.28 ± 0.38	35.74 ± 2.42
5	9.0	4.5	2.0	234.51 ± 0.90	0.17 ± 0.01	−3.42 ± 0.22	72.83 ± 1.32
6	9.0	3.5	2.5	302.81 ± 1.94	0.23 ± 0.02	−3.82 ± 0.25	49.64 ± 2.21
7	8.0	4.5	2.5	242.52 ± 3.51	0.16 ± 0.01	−2.76 ± 0.50	70.01 ± 3.23
**8**	**9.0**	**4.5**	**2.5**	**247.22 ± 2.77**	**0.17 ± 0.03**	**−3.34 ± 0.42**	**90.12 ± 1.15**

Data are represented as mean ± SD (*n* = 3).

**Table 2:** Four different spray drying conditions of PGZ-NPs after resuspension in water.

Formulation	Size (nm)	PI	ZP (mV)	EE (%)
Spray 1	366.61 ± 13.48	0.39 ± 0.01	−6.93 ± 1.04	90.05 ± 1.14
Spray 2	271.31 ± 2.78	0.24 ± 0.00	−7.57 ± 1.53	89.13 ± 2.21
Spray 3	328.02 ± 1.34	0.29 ± 0.03	−6.48 ± 0.31	90.10 ± 1.54
Spray 4	276.64 ± 1.75	0.23 ± 0.00	−9.87 ± 0.48	88.92 ± 1.21

## Data Availability

Not applicable.

## Supplementary Materials

The following are available online at https://www.mdpi.com/article/10.3390/pharmaceutics13111751/s1, Figure S1. TEM image of Spray 2 formulation once resuspended in water. Table S1. Release kinetic parameters of PGZ-NPs before and after spraying.
