# Supplementary material for: Stabilization by Nano Spray Dryer of Pioglitazone Polymeric Nanosystems: Development, In Vivo, Ex Vivo and Synchrotron Analysis
Source: Pharmaceutics. 2021 Oct 20;13(11):1751. doi: 10.3390/pharmaceutics13111751 (PMC8617923; doi:10.3390/pharmaceutics13111751)
Supplement: Supplementary file 1 [file pharmaceutics-13-01751-s001.zip › pharmaceutics-1407875 supplementary updated.pdf]

pharmaceutics
MDPI

Supplementary Materials: Stabilization by Nano Spray Dryer of Pioglitazone Polymeric Nanosystems: Development, In Vivo, Ex Vivo and Synchrotron Analysis.

Marcelle Silva-Abreu*, Esther Miralles, Christina S. Kamma-Lorger, Marta Espina, Maria Luisa García and Ana Cristina Calpena

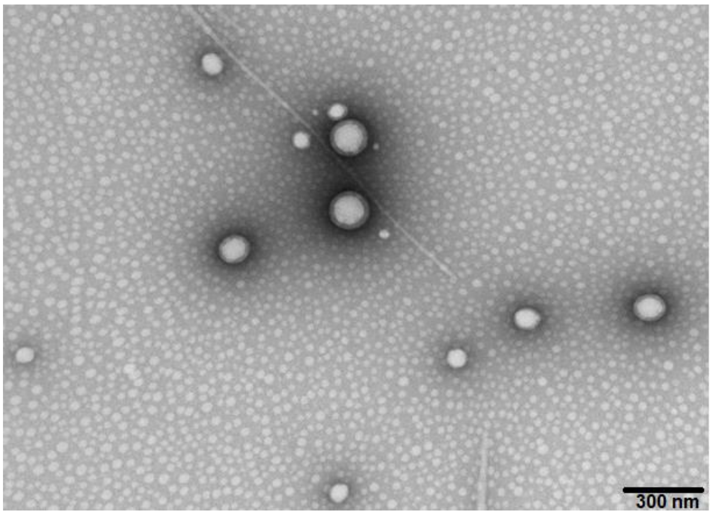

Figure S1. TEM image of Spray 2 formulation once resuspended in water.

Table S1. Release kinetic parameters of PGZ-NPs before and after spraying.

Kinetics Models	Equation	Parameters	Before spray	
			PGZ-NPs	Free PGZ
One exponential association	$Q_t = Q_\infty \cdot (1 - e^{-K \cdot t})$	AIC	-	16.79
		r^2	-	0.99
Korsmeyer Peppas	$Q_t = K \cdot t^n$	AIC	13.20	-
		r^2	0.99	-
			After spray	
One exponential association	$Q_t = Q_\infty \cdot (1 - e^{-K \cdot t})$	AIC	Spray 1	64.85
		r^2		0.99
Korsmeyer Peppas	$Q_t = K \cdot t^n$	AIC	Spray 2	46.02
		r^2		0.99
One exponential association	$Q_t = Q_\infty \cdot (1 - e^{-K \cdot t})$	AIC	Spray 3	65.94
		r^2		0.99
Hyperbola	$Q_t = Q_\infty \cdot t/(Kd + t)$	AIC	Spray 4	43.66
		r^2		0.99

Q_t = cumulative amount of drug release at time t; Q_∞= maximum amount of drug released; K_0, K = release rate constants; t = time in hours; Kd = dissolution time; n is the diffusion release exponent that can be used to characterize the different release mechanisms; r^2 = determination coefficient; *AIC* = Akaike's information criterion.
